# Load management in elite German distance runners during 3‐weeks of high‐altitude training

**DOI:** 10.14814/phy2.12845

**Published:** 2016-06-29

**Authors:** Billy Sperlich, Silvia Achtzehn, Markus de Marées, Henning von Papen, Joachim Mester

**Affiliations:** ^1^ Integrative and Experimental Training ScienceInstitute of Sport ScienceUniversity of WürzburgWürzburgGermany; ^2^ Institute of Training Science and Sport InformaticsGerman Sport UniversityCologneGermany; ^3^ The German Research Centre of Elite SportGerman Sport UniversityCologneGermany; ^4^ German Athletics AssociationDarmstadtGermany

**Keywords:** Blood urea nitrogen, creatine kinase, hematocrit, hemoglobin concentration, monitoring, POCT, red blood cell count, white blood cell count

## Abstract

There is a debate on the optimal way of monitoring training loads in elite endurance athletes especially during altitude training camps. In this case report, including nine members of the German national middle distance running team, we describe a practical approach to monitor the psychobiological stress markers during 21 days of altitude training (~2100 m above sea‐level) to estimate the training load and to control muscle damage, fatigue, and/or chronic overreaching. Daily examination included: oxygen saturation of hemoglobin, resting heart rate, body mass, body and sleep perception, capillary blood concentration of creatine kinase. Every other day, venous serum concentration of blood urea nitrogen, venous blood concentration of hemoglobin, hematocrit, red and white blood cell were measured. If two or more of the above‐mentioned stress markers were beyond or beneath the athlete's normal individual range, the training load of the subsequent training session was reduced. Running speed at 3 mmol L^−1^ blood lactate (V_3_) improved and no athlete showed any signs of underperformance, chronic muscle damage, decrease body and sleep perception as well as activated inflammatory process during the 21 days. The dense screening of biomarkers in the present case study may stimulate further research to identify candidate markers for load monitoring in elite middle‐ and long‐distance runners during a training camp at altitude.

## Introduction

Elite endurance athletes frequently aim to enhance performance by executing training camps with increased training load (i.e., frequency, duration, and intensity) at sea‐level or at altitude inducing significant muscular stress with the potential risks for tissue damage (Meeusen et al. 2013) and impaired immune health (Walsh et al. 2011). The individual monitoring of training loads, especially during high‐altitude training (Chapman 2013)**,** is essential for optimizing adaptation and performance as well as reducing the risk of chronic fatigue (Kiely 2012; Halson 2014).

A recent review concluded that particularly at altitude, (1) some athletes struggle compared to others when exposed to hypoxia; and (2) cautious screening may aid to identify such athletes (Chapman 2013). In this context, several objective and subjective stress markers have been proposed to monitor the individual training load at sea‐level and altitude (Banfi et al. 2012; Chapman 2013; Halson 2014), thereby, securing optimal adaptation and counteracting unwanted side effects such as overtraining symptoms. However, not every biomarker resembles as legitimate or sensitive variable for monitoring the stress of training (Halson 2014). Sophisticated biochemical analysis (e.g., muscle biopsies, special blood analysis) is invasive, cost worthy, depends on a laboratory and takes too long time to analyze in order to react timely (i.e., reducing volume and/or exercise intensity in subsequent training session) when chronic fatigue occurs. Therefore, from a practical point of view, subjective and objective biomarkers should be assessed in a set of markers and each marker should be assessed effortlessly and with rapid reporting of simple, yet scientifically trustworthy, feedback. From this perspective, point‐of‐care‐testing (POCT) allows simple assessment of biomedical parameters which can be performed at the bedside or on the training site providing convenient and immediate information to the athlete. In the present case study, we intended (1) to individually estimate the training response and to counteract chronic overreaching by applying promising POCT‐derived biomarkers; and (2) to discuss the practical applications of these variables for monitoring training load during 3 weeks of altitude training from elite German middle‐ and long‐distance runners.

## Case Report

Nine members (7 male and 2 female) of the German middle‐ and long‐distance national team (age: 22 ± 3 years; size: 181.1 ± 8.9 cm; body mass: 67.2 ± 10.8 kg; the individual performance is summarized in Table 1) performed a 21 day altitude training camp (~2100 m above sea‐level in Flagstaff, AZ) after a west‐bound flight with approximately 20–23 h of air and car transportation including a time shift of 9 h.

**Table 1:** Performance of the athletes

Athlete	400 m [s]	800 m [min:s]	1.500 m [min:s]	3.000 m [min:s]	5.000 m [min:s]	10.000 m [min:s]	Half Marathon [h:min:s]	Marathon [h:min:s]
1		<1:47	<3:44					
2	<47.9	<1:47						
3		<1:51	<3:42					
4			<3:44	<8:10				
5					<16:15	<32:50	<1:14:40	<2:28:30
6						<29:35	<1:04:50	<2:16:10
7		<1:49						
8			<4:12	<9:09	<16:10			
9			<3:42					

The training camp was designed as a preseason preparation block. Two of the athletes participated in middle distance Olympic events and the others in national or international races. All procedures were in accordance with the declaration of Helsinki and the institute's ethical board, and all athletes gave their consent to participate in this study.

During the morning routine, all athletes reported to a field laboratory between 7 and 9 am in a fasting condition. Daily examination included: resting oxygen saturation of hemoglobin (typical error measurement (TEM): ≤1.5%) and resting heart rate (TEM: ≤2.5%; Masimo Rad‐5V Pulse Oximeter), body mass (TEM: ≤1.0%; Tanita BC 418 MA, Tokio, Japan), self‐reported body and sleep perception (1–6 scale, one being perfect), capillary blood concentration of creatine kinase (TEM: ≤3.8%; Spotchem EZ Sp 4430, Arkray, Kyoto, Japan). Every other day, venous serum concentration of blood urea nitrogen (TEM: ≤3.1%; Spotchem EZ Sp 4430) as well as venous blood concentration of hemoglobin and hematocrit (TEM: ≤1.5% and ≤2.0%), red and white blood cell count (TEM: ≤2.0% and ≤3.5%; Sysmex KX 21‐N, Sysmex, Kobe, Japan) were assessed. No data were obtained on day 12 due to a day‐long desert hike (Sperlich et al. 2010a).

At day 4 and 21, all athletes performed an incremental field step tests (4 × 2000 m with an initial speed of 4.0 m sec^−1^ and increase: 0.2 m sec^−1^ per interval) on a 400 m track to assess running speed at 3 mmol L^−1^ blood lactate (V_3_; TEM: ≤1.0%; Biosen C_line, EKF Diagnostics, Germany). V_3_ was calculated by linear extrapolation, using the lactate concentration at the running velocities directly before and after the achievement of 3 mmol L^−1^ capillary blood lactate concentration.

The training program for all 21 days is summarized in Table 2.

**Table 2:** Training volume and intensity of all morning and afternoon training sessions without warm‐up

Day	Morning session	Afternoon session	Training time [min]	Volume [km]
0	Arrival	Assembly of field laboratory		
1	40 min easy jogging	40 min at 70% of V_3_	80	7.5
2	2 h hiking	10 km at 80% of V_3_	180	18
3	10 km at 82% of V_3_	2 h hiking	180	18
4	4 × 2000 m incremental test	Core strength	180	12
5	18 km at 82% of V_3_		120	18
6	10 km at 82% of V_3_	10 × 200 m uphill intervals at <85% race pace with 2 min recovery (walking or easy jogging)	150	18
7	8 × 1000 m at 100–105% of V_3_	45 min at 70% of V_3_	150	17
8	15 km at 83% of V_3_	Core strength	90	16
9	15 km at 83% of V_3_	Core strength	180	17
10	1 h at 80% of V_3_	1 h at 82% of V_3_	180	26
11	10 km at V_3_	45 min at 70% of V_3_	150	19
12	Hiking Grand Canyon		500	33
13	1 h 83%V3	10 × 200 m uphill intervals at 88% race pace with 2 min recovery (walking or easy jogging)	150	18
14	8 × 1000 m 105–110% of V_3_	45 min at 70% of V_3_	150	17
15	15 km at 83% of V_3_		90	16
16	10–12 km at 87% of V_3_	Core strength	180	13
17	1 h at 82% of V_3_		90	14
18	10 km at V_3_	45 min at 70% of V_3_ and core strength	150	19
19	15–20 km at 83% of V_3_		72	15
20	1 h at 82% of V_3_	10 × 200 m uphill intervals at 90% race pace with 2 min recovery (walking or easy jogging)	150	18
21	4 × 2000 m incremental test	45 min at 70% of V_3_	180	14
22	Departure			

V_3_ = Running speed corresponding to 3 mmol L^−1^ of blood lactate.

As a general training rule, training load (volume and/or exercise intensity) was reduced when two or more of the above‐mentioned stress markers were outside the normal individual range of the athlete. We did not record the specific training adjustments since they occur frequently and are based on the coaches’ and athletes’ experience. The normal individual ranges (mean ± standard deviation) for each variable and each athlete were quantified based on the values derived from the data of day 1 and/or matched with the individual data obtained from previous monitoring. All mean ± SD data for each variable and each day of all athletes during 3‐weeks altitude training are presented in Table 3.

**Table 3:** Mean ± SD data for each variable and each day of all athletes during 3‐weeks altitude training

	1	2	3	4	5	6	7	8	9	10	11	12	13	14	15	16	17	18	19	20
Oxygen saturation [%]																				
Mean	96.8	97.0	96.1	96.2	95.8	95.9	96.2	96.9	95.7	96.7	95.4		97.4	97.2	97.4	96.2	97.0	96.4	96.6	96.4
SD	0.8	1.2	1.4	1.4	1.1	0.9	1.0	1.5	0.9	1.1	1.5		1.4	1.4	1.3	1.4	1.4	1.1	0.9	1.9
Heart rate at rest [bpm]																				
Mean	58.3	60.9	54.6	62.7	57.6	59.3	54.6	54.2	51.0	57.1	51.3		49.2	52.1	48.0	49.3	48.2	50.2	48.1	50.3
SD	9.5	11.1	10.7	8.6	10.3	12.0	7.7	7.1	6.2	8.6	5.8		9.7	11.8	6.5	8.3	4.9	7.6	5.9	5.2
Body mass [kg]																				
Mean	66.3	66.7	66.7	66.6	66.6	66.7	66.3	66.7	66.5	66.9	66.7		66.5	66.6	66.7	66.7	66.6	66.7	66.6	66.6
SD	10.8	10.7	10.6	10.9	10.4	10.6	10.6	10.8	10.6	11.0	10.6		10.4	10.5	10.5	10.5	10.2	10.6	10.7	10.3
Quality of sleep [a.u.]																				
Mean	2.1	2.5	2.6	1.8	2.9	2.0	2.6	2.9	2.4	2.3	2.3		2.2	2.7	2.9	2.0	2.9	2.6	2.3	2.8
SD	0.8	0.5	0.5	1.0	1.5	1.0	0.9	1.2	0.9	0.5	1.0		1.1	1.1	1.3	1.0	1.8	0.7	0.5	1.3
Body perception [a.u.]																				
Mean	2.7	2.4	2.6	2.8	2.8	2.6	2.8	2.9	2.6	2.3	2.6		2.7	2.3	2.6	2.3	2.5	2.4	2.6	3.0
SD	0.8	0.5	0.7	1.0	0.5	0.7	0.5	0.6	0.5	0.5	0.5		0.9	0.5	0.5	0.5	0.5	0.5	1.0	1.0
Creatine kinase [U L^−1^]																				
Mean	182.2	260.6	321.3	372.2	395.3	449.2	366.6	314.9	451.8	405.1	413.3		271.9	351.2	331.2	262.9	450.0	434.7	341.1	336.7
SD	75.9	112.7	125.5	119.5	141.4	155.0	140.4	110.2	183.6	200.4	174.3		107.9	169.7	137.0	108.4	166.7	197.9	151.1	180.9
Blood urea nitrogen [mmol L^−1^]																				
Mean	4.2		4.5		4.8		5.0		5.0		4.1				3.8		3.8		3.7	
SD	1.8		1.0		0.8		1.6		1.2		0.9				1.0		0.7		1.0	
Hemoglobin [g·dL‐1]																				
Mean	16.4		16.1		16.6		16.3		16.0		16.0		16.4		16.4		16.3		16.5	
SD	0.7		1.0		0.8		0.5		0.8		0.8		0.9		0.7		1.0		0.9	
Hematocrit [%]																				
Mean	47.7		46.0		47.9		46.8		45.4		45.6		46.7		47.1		46.2		47.3	
SD	1.7		2.5		2.0		1.6		2.1		1.9		2.2		1.7		2.5		2.6	
Red blood cells count [×10e6 *μ*L^−1^]																				
Mean	5.5		5.3		5.6		5.4		5.4		5.4		5.4		5.4		5.6		5.4	
SD	0.3		0.4		0.4		0.3		0.4		0.3		0.5		0.4		0.4		0.5	
White blood cells count [×10e6 *μ*L^−1^]																				
Mean	6.0		5.8		6.2		5.6		5.5		6.0		5.5		5.6		6.3		6.4	
SD	0.9		0.9		0.8		1.1		0.8		1.0		0.9		0.8		0.9		1.8	
Running speed at 3 mol L^−1^ (V3) blood lactate [m sec^−1^]																				
Mean				4.50																4.63
SD				0.30																0.27

**Figure 1:**
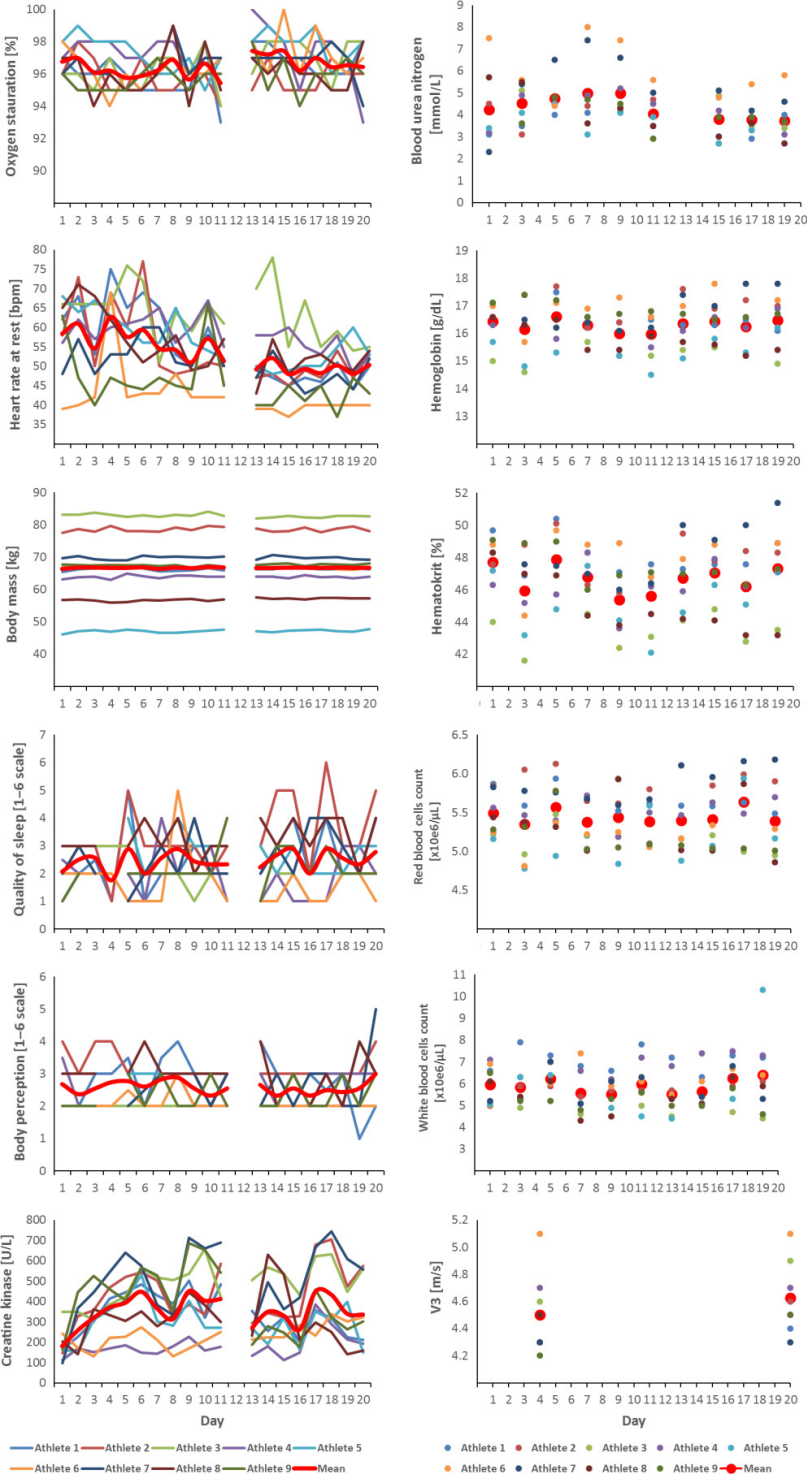
The mean (red line or dot) and individual day‐to‐day variation of different stress and performance markers of nine elite athletes during a 21 day training camp.

In the present case study, V_3_ increased from day 4 to day 21 (4.4 ± 0.3 to 4.6 ± 0.3 m sec^−1^). None of the athletes showed or reported any signs of underperformance, chronic muscle damage, decreased body and sleep perception during the 21 days of exercise. Only athlete 3 showed signs of inflammation (elevated WBC; Fig. 1) on day 19 following an accident involving widespread open leg wounds.

## Discussion

It is well known that persistent exposure to hypoxia and increased training loads have detrimental effects on body mass (Westerterp and Kayser 2006), muscle architecture (Howald and Hoppeler 2003), and exercise capacity (Baquet et al. 2002), however, none of our athletes showed any loss in body mass nor exercise capacity. In fact, the V_3_ slightly improved over time which could be due to metabolic adaptation or a result of a phenomenon called lactate paradox (a yet unclear observation showing lower‐than‐expected amounts of lactate) when exposed to hypoxia (Hochachka et al. 2002).

Additionally, a rapid elevation in volume and/or exercise intensity for a sustained period, as designed in this study, has the potential to result in “overreaching” or symptoms related to overtraining (Baquet et al. 2002; Smith 2003; Schmitt et al. 2006). This may lead to reduced maximum physical capacity (Baquet et al. 2002) or chronic performance decrements including chronic fatigue symptoms (i.e., exhausted feeling, tiredness, lack of energy with impaired sleep, lower immunity, or inflammation processes) (Montpetit et al. 1987; Ryan et al. 1987; Sperlich et al. 2010b).

Usually, stress markers such as creatine kinase, urea, hemoglobin concentration, hematocrit, red and white blood cells are measured in longer time intervals or when underperformance occurs. However, in training camps with demanding training loads, it is paramount to timely counteract negative side effects such as chronic muscle damage or underperformance. Consequently, during training camps, easy and fast determinable parameters should be assessed on a daily basis in order to control training load. CK and blood urea nitrogen are a blood‐borne biomarkers (easily measured with POCT with rapid feedback to the athlete) and have shown to accurately reflect changes in fatigue during a training camp (Hecksteden et al. 2016). Blood cell counts and measuring of hemoglobin concentration cannot detect overreaching or underperformance per se; however, these variables are helpful in providing information on the actual health status of the athlete (Robson‐Ansley et al. 2009) and should be frequently assessed to ensure a good health status.

Based on the current data, the mean heart rate decreased from Day 1 to the end of the training camp, which is a normal sign during altitude exposure (Mazzeo 2008) as well as of positive aerobic training adaptations. However, the interpretation of repeated measures of heart rate at rest (Buchheit 2014) over time may be complex since the heart rate is influence by cardiac structure, plasma volume, autonomic activity, age, body position, and oxygen partial pressure.

Additionally, continuous exposure to hypoxia has opposing effects on mental functioning and quality of sleep (Weil 2004), and together can negatively impact the quality of a training and general well‐being. Although none of our athletes reported overtraining symptoms, several athletes complained of impaired sleep which is a well‐known side effect among athletes in altitude training camps. For this reason, simple questionnaires with fatigue and sleep‐related scales represent simple and inexpensive measures to estimate the training load and subsequent responses to training. However, questionnaires and fatigues scales rely on subjective information, which need to be verified with physiological data, as pointed out earlier (Borresen and Lambert 2009).

For this reason, during long‐term diagnostic, we defined individual ranges for all subjective and objective variables. In the case two or more of the above‐mentioned stress markers were beyond or beneath the normal range for the athletes, training load (i.e., intensity or volume) was reduced the subsequent day. This procedure was based on experiences obtained from numerous training interventions and recommendations summarized previously (Halson 2014). From a scientific point of view, we cannot be sure whether this procedure may be superior to other marker sets or data interpretation, however, from a practical point of view, this procedure allowed us (1) to improve V_3_; and (2) timely counteract signs of possible chronic overreaching with no signs of activated inflammatory process (except for athlete 3 on day 19) and loss of sleep.

Some limitations of this case study are noteworthy. Although the training load adjustments were based on our screening including the judgment and experience of the coach and the athlete, we cannot prove that the performance was affected by the change in training load. Potentially no athlete would have developed symptoms, even if the training load was unaltered. Since we did not include a control group, we cannot confirm if the procedures presented here were superior to others to improve performance. However, a different study design including a control group involving elite‐level athletes is practically impossible since novel training modifications might not result in performance gains or might even lead to overtraining symptoms.

The adjustment of intensity and volume in elite runners by screening of selected biomarkers as described in the present case study may stimulate further research to identify candidate markers for load monitoring in elite middle‐ and long‐distance runners during a high‐altitude training camp.

## Conflict of Interest

None declared.
